# *Halomonas gemina* sp. nov. and *Halomonas llamarensis* sp. nov., two siderophore-producing organisms isolated from high-altitude salars of the Atacama Desert

**DOI:** 10.3389/fmicb.2023.1194916

**Published:** 2023-06-12

**Authors:** Christian Hintersatz, Shalini Singh, Luis Antonio Rojas, Jerome Kretzschmar, Sean Ting-Shyang Wei, Khushal Khambhati, Sabine Kutschke, Falk Lehmann, Vijai Singh, Rohan Jain, Katrin Pollmann

**Affiliations:** ^1^Department of Biotechnology, Helmholtz Institute Freiberg for Resource Technology, Helmholtz-Zentrum Dresden-Rossendorf, Dresden, Germany; ^2^Department of Chemistry, Universidad Católica del Norte, Antofagasta, Chile; ^3^Department of Actinide Thermodynamics, Institute of Resource Ecology, Helmholtz-Zentrum Dresden-Rossendorf, Dresden, Germany; ^4^Department of Biogeochemistry, Institute of Resource Ecology, Helmholtz-Zentrum Dresden-Rossendorf, Dresden, Germany; ^5^Department of Biosciences, School of Science, Indrashil University, Mehsana, India

**Keywords:** halophilic bacteria, siderophores, polyphasic taxonomic, desferrioxamine E, Atacama Desert

## Abstract

**Introduction:**

This study aimed to identify and characterize novel siderophore-producing organisms capable of secreting high quantities of the iron-binding compounds. In the course of this, two not yet reported halophilic strains designated ATCHA^T^ and ATCH28^T^ were isolated from hypersaline, alkaline surface waters of Salar de Llamará and Laguna Lejía, respectively. The alkaline environment limits iron bioavailability, suggesting that native organisms produce abundant siderophores to sequester iron.

**Methods:**

Both strains were characterized by polyphasic approach. Comparative analysis of the 16S rRNA gene sequences revealed their affiliation with the genus *Halomonas*. ATCHA^T^ showed close similarity to *Halomonas salicampi* and *Halomonas vilamensis*, while ATCH28^T^ was related closest to *Halomonas ventosae* and *Halomonas salina*. The ability of both strains to secrete siderophores was initially assessed using the chromeazurol S (CAS) liquid assay and subsequently further investigated through genomic analysis and NMR. Furthermore, the effect of various media components on the siderophore secretion by strain ATCH28^T^ was explored.

**Results:**

The CAS assay confirmed the ability of both strains to produce iron-binding compounds. Genomic analysis of strain ATCHA^T^ revealed the presence of a not yet reported NRPS-dependant gene cluster responsible for the secretion of siderophore. However, as only small amounts of siderophore were secreted, further investigations did not lie within the scope of this study. Via NMR and genomic analysis, strain ATCH28^T^ has been determined to produce desferrioxamine E (DFOE). Although this siderophore is common in various terrestrial microorganisms, it has not yet been reported to occur within *Halomonas*, making strain ATCH28^T^ the first member of the genus to produce a non-amphiphilic siderophore. By means of media optimization, the produced quantity of DFOE could be increased to more than 1000 µM.

**Discussion:**

Phenotypic and genotypic characteristics clearly differentiated both strains from other members of the genus *Halomonas*. Average nucleotide identity (ANI) values and DNA–DNA relatedness indicated that the strains represented two novel species. Therefore, both species should be added as new representatives of the genus *Halomonas*, for which the designations *Halomonas llamarensis* sp. nov. (type strain ATCHA^T^ = DSM 114476 = LMG 32709) and *Halomonas gemina* sp. nov. (type strain ATCH28^T^ = DSM 114418 = LMG 32708) are proposed.

## 1. Introduction

Siderophores are a large group of small-sized, iron-chelating compounds, which are excreted by a plethora of microorganisms in order to sequester iron from environments with low iron bioavailability. Due to their structural diversity and their ability to bind to various metals, those biomolecules are promising compounds for utilization in future technologies, such as biosensors (Nosrati et al., 2018), the selective recovery of metals from low-concentrated wastewaters (Jain et al., 2019), or selective bioleaching (Williamson et al., 2021). However, typically only small amounts of the compounds are secreted by the producing organism, making their utilization often not viable. At the same time, the low commercial availability of siderophores as well as the high prices of the compounds hinders progress in this field. Hence, organisms producing higher amounts of siderophores are a sought-after asset. To this day little is reported about siderophore-producing organisms native to hypersaline, alkaline environments. As the bioavailability of iron is substantially lower above neutral pH, organisms originating from this environment might possess the ability to produce greater amounts of the metal-binding compounds and therefore were focused on for this study.

*Halomonas* is a genus known for its high metabolic versatility, e.g., the ability to produce exopolysaccharides (Bouchotroch et al., 2001; Poli et al., 2013), denitrify (Peyton et al., 2001; Yoshie et al., 2006), or degrade aromatic compounds (García et al., 2004; Chmura et al., 2008) makes them a valuable resource for biotechnological applications. One of those might be the high-yield production of siderophores. The genus *Halomonas* is part of *Halomonadaceae*, a comparatively large family that belongs to the class *Gammaproteobacteria*. It was originally proposed by Franzmann et al. (1988) in order to accommodate the genera *Deleya*, *Halovibrio*, and *Halomonas*. To this date, it comprises 14 validly published genera, i.e., *Aidingimonas*, *Carnimonas*, *Chromohalobacter*, *Cobetia*, *Halomonas*, *Halotalea, Halovibrio, Kushneria*, *Larsenimonas*, *Modicisalibacter*, *Pistricoccus*, *Salinicola*, *Terasakiispira*, and *Zymobacter*.^1^ The type genus *Halomonas* was established by Vreeland and co-workers with the description of *Halomonas elongata* in 1980 (Vreeland et al., 1980). At the time of writing, the genus *Halomonas* includes 121 recognized species, making it by far the largest group within the family.^2^ Members of the genus are Gram-stain-negative, rod-shaped, chemo-organotrophic, non-endospore-forming, catalase positive, aerobic or facultative anaerobic and motile by means of lateral, polar or peritrichous flagella. They are slightly to moderately halophilic organisms with the ability to grow at salt concentrations between 0.1–35.5% (w/v). Chemotaxonomically they are characterized by the presence of ubiquinone 9 as the main respiratory quinone and C_16:0_, C_18:1_ ω7c, C_16:1_ ω7c, C_12:0_ 3-OH, and C_19:0_ cyclo ω8c as typical major cellular fatty acids (Ventosa et al., 2021). Halomonads are widely distributed within saline environments, such as hypersaline lakes (Xu et al., 2007; Lu et al., 2020), salt mine soils (Wang et al., 2008, 2014), seawaters (Yoon et al., 2001; Xu et al., 2013), solar salterns (Lim et al., 2004; Lee et al., 2005), soda lakes (Duckworth et al., 2000; Boltyanskaya et al., 2007), or fermented seafood (Yoon et al., 2002; Jeong et al., 2013). As the genus is large and their natural habitats often iron-limited, various different types of siderophores have been reported to be produced by members of *Halomonas*. Those siderophores are aquachelins (Martinez et al., 2000), loihichelins (Homann et al., 2009), potashchelins (Li et al., 2020), halochelins (Figueroa et al., 2015), as well as sodachelins (Serrano Figueroa et al., 2015), all of which are of amphiphilic character.

This study aimed to find novel siderophore-producing organisms with the ability to produce high amounts of the metal-binding compounds. In pursuit of this, two yet unknown species of the genus *Halomonas*, designated ATCHA^T^ and ATCH28^T^, were isolated from hypersaline, alkaline lagoons of the Atacama Desert. The strains’ taxonomic status was determined utilizing a polyphasic approach. Both isolates were further investigated for their siderophore biosynthetic gene cluster and production of respective siderophores. Via genomic analysis, NMR, and HPLC, strain ATCH28^T^ was found to synthesize high amounts of the siderophore desferrioxamine E (DFOE), a well-studied siderophore, which is commonly produced by terrestrial organisms. This makes it the first member of the genus to produce a non-amphiphilic siderophore, as well as the first member of the genus reported to produce this siderophore. Subsequently, the effect of various media components on DFOE biosynthesis was investigated in an attempt to further increase the amounts of siderophore produced.

## 2. Materials and methods

### 2.1. Cultivation and isolation of siderophore-producing organisms

The strains ATCHA^T^ and ATCH28^T^ were isolated from the surface waters of Salar de Llamará (21°21′28”S 69°35′56”W) and Salar de Ascotán (21°36′45”S 68°18′12”W), respectively, both of which are located in the Atacama Desert of northern Chile. The environmental samples of Salar de Llamará were gathered on 12 February 2020, while the samples originating from Salar de Ascotán were taken on 20 January 2020. The water samples were analyzed using inductively coupled plasma mass spectrometry (ICP-MS) and anion-chromatography in order to determine the concentration of commonly occurring cat-and anions. Subsequently, two growth media were designed with the aim to mimic the conditions present at the isolation sites. The isolation of ATCHA^T^ was carried out using IM4 (per liter: 0.1 g LiCl, 0.8 g CaCl_2_ × 2 H_2_O, 8 g MgSO_4_ × 7 H_2_O, 130 g NaCl, 2.6 g K_2_SO_4_, 30 g Na_2_SO_4_, 2.5 g casamino acids), while strain ATCH28^T^ was isolated in IM1 (per liter: 0.3 g LiCl, 0.8 g CaCl_2_, 81.8 g MgSO_4_ × 7 H_2_O, 40 g NaCl, 3 g K_2_SO_4_, 30 g Na_2_SO_4_, 2.5 g casamino acids), both of which were adjusted to pH 8 using 1 M NaOH. Aliquots of 100 μL of a 10-fold serial dilution of both samples in sterile 10% (w/v) NaCl were plated on solidified IM1 or IM4 (1.5% (w/v) agar). Consequently, the picked colonies were grown in liquid cultures and further purified three times by means of sub-cultivation on solidified isolation medium. Induction of siderophore production was achieved by means of iron-limitation in the isolation media and tested via liquid chromeazurol S (CAS) assay (Alexander and Zuberer, 1991). The strains were preserved in IM1 or IM4, supplemented with 30% (v/v) glycerol, and stored at-80°C.

### 2.2. Genomic analysis

Phylogenetic studies of strains ATCHA^T^ and ATCH28^T^ were conducted on the foundation of 16S rRNA gene sequences. The extraction of genomic DNA was achieved via the Nukleospin DNA RapidLyse kit (Macherey-Nagel) following the manufacturer’s instructions. Thereafter, the 16S rRNA genes of both strains were amplified via PCR utilizing the universal primers 7F (5’-AAGASTTTGATYNTGGCTCAG-3′) and 1513R (5’-TACGGYTACCTTGTTACGACTT-3′). The amplified PCR products were purified with the MSB^®^ Spin PCRapace kit (Inviter) and the sequences determined by Sanger sequencing. The 16S rRNA gene sequences were compared with reference sequences of the GenBank database via Nucleotide BLAST. In MEGA 11 (Tamura et al., 2021), the multiple sequence alignment program MUSCLE (Edgar, 2004) was used in order to align the 16S rRNA gene sequences of the herein described species with reference sequences of closely related species and a phylogenetic tree was inferred using the maximum-likelihood algorithm and Tamura 3-parameter model (Tamura, 1992). The assessment of the tree topology’s confidence level was done by bootstrap resampling method based on 2000 replicons (Felsenstein, 1985).

The draft genome sequences of strains ATCHA^T^ and ATCH28^T^ were obtained by HiSeq sequencer (250 bp, pair-end). Using Trimmomatic 0.40 (Bolger et al., 2014), low-quality raw reads (QS < 30) were trimmed and the resulting reads assembled in SPAdes 3.15.3 (Bankevich et al., 2012) employing the genomes of *H. salicampi* BH103 for ATCHA^T^ and *H. ventosae* Al12 for ATCH28^T^ as reference. The calculations of the genomes’ general statistics, such as the number of contigs, N50 and G + C content (%) were conducted with QUAST (Gurevich et al., 2013). Annotation of the gene coding regions with putative functions was done by the NCBI Prokaryotic Genome Annotation Pipeline (Tatusova et al., 2016). In order to extract and estimate contaminations of the 16S rRNA gene sequences within the genomes, ContEst16S (Lee et al., 2017) was utilized. CheckM (Parks et al., 2015) was applied to evaluate the completeness of the genomes as well as contaminations based on the presence and duplication of single-copy marker genes. Digital DNA–DNA hybridization values (dDDH) between ATCHA^T^ or ATCH28^T^ and closely related species were determined with the Genome-to-Genome Distance Calculator (GGDC 3.0; Meier-Kolthoff et al., 2022). Average Nucleotide Identities (ANI) were calculated using the OrthoANIu algorithm (Yoon et al., 2017) provided by the EZBioCloud web service.

The phylogenomic analysis was conducted using the UBCG2 pipeline (Kim et al., 2021) with RAxML for phylogeny reconstruction. This pipeline uses a set of 81 single-copy core genes commonly present in all bacterial genomes.^3^ The robustness of the nodes was estimated from the gene support index (GSI) with a value of 100. Other parameters were set as default.

Additionally, the draft genomes were investigated for the presence of siderophore biosynthetic gene clusters using the antibiotics and secondary metabolite analysis shell (AntiSMASH) 6.0 online tool (Blin et al., 2021).

### 2.3. Phenotypic characterization

For the investigation of morphological characteristics of strains ATCHA^T^ and ATCH28^T^, cells were grown in liquid medium at 30°C for 24 h and observed using phase-contrast microscopy (BX43, Olympus). In order to visualize the cells’ flagellation, the Leifson stain method was utilized (Piccolomini et al., 1999). The strains’ capacities to grow under anaerobic conditions were tested on solidified medium, which was supplemented with 1 g/L NaNO_3_ and incubated at 30°C in an anaerobic jar using the Anaerocult^™^ A system (Merck) for the generation of an anaerobic atmosphere. The determination of growth range and optimal conditions for temperature, pH and salinity was conducted employing a medium containing 5 g/L peptone, 1 g/L yeast extract and 0.1 g/L ferric citrate. For the determination of salt tolerance and optimal concentration for growth, the quantity of NaCl in the medium was adjusted to 0, 0.5, 1, 3, 5, 7, 10, 12, 15, 17 or 20% (w/v) and experiments were carried out at pH adjusted to 8 with 1 M NaOH and an incubation temperature of 30°C. Temperatures and pH values tested were 4, 20, 30, 40 or 50°C and 5.5, 6, 6.5, 7, 7.5, 8, 8.5, 9, or 10, respectively, with a salt concentration of 100 g/L NaCl. The pH was adjusted to 8.0 for temperature optimization experiments, while the temperature was set to 30°C for pH optimization experiments. In order to stabilize the pH values for the determination of range and optima, the medium was supplemented with 0.1 M of MES (pH 5.5–6.5), HEPES (pH 7–8), or CAPSO (pH 8.5–10). All growth experiments were carried out for 3 days and monitored by means of change in optical density. The abilities of ATCHA^T^ and ATCH28^T^ to utilize a variety of substrates as sole carbon sources were tested using Biolog Gen III plates with a suspension medium containing 2 g/L NH_4_Cl, 2 g/L MgSO_4_ × 7 H_2_O, 0.1 g/L CaCl_2_, 0.5 g/L KH_2_PO_4_, 100 g/L NaCl and 0.01% (w/v) tetrazolium chloride. Wells that developed a purple coloration due to the reduction of tetrazolium chloride to formazan after 7 days of growth at 30°C were deemed as positive. Oxidase and catalase activities of the strains were tested following the protocols described by Smibert and Krieg (1994), while the hydrolyzation of casein, gelatin, starch and DNA was determined following the methods described by Høvik Hansen and Sørheim (1991). Production of H_2_S from cysteine was tested via lead acetate paper (Supelco). To further analyze the biochemical properties of both strains, API 20NE test kits (bioMérieux) were used according to the manufacturer’s instructions, except for the usage of 10% saline solution for the preparation of cell suspensions and supplementation of the provided AUX medium with 10% NaCl. Enzymatic activities of ATCHA^T^ and ATCH28^T^ were elucidated by means of the API ZYM test kit (bioMérieux) following the included instructions.

### 2.4. Chemotaxonomic characterization

The determination of respiratory quinones as well as cellular fatty acids of strains ATCHA^T^ and ATCH28^T^ was carried out by the identification service of the German collection of microorganisms and cell cultures (DSMZ) (Braunschweig, Germany). Production of biomass was carried out in the respective isolation media at 30°C. The cells were harvested in exponential growth phase, washed in sterile 7% NaCl and subsequently lyophilized.

### 2.5. Production and purification of siderophores

For siderophore production, the bacteria were grown in glass bioreactors with 5 L working volume. The pH was set to 8 and regulated constantly with 2 M NaOH, the temperature was kept at 30°C and airflow adjusted to 1.5 L/min. After 7 days the culture medium was centrifuged at 10000 g for 20 min and the siderophore concentration in the supernatant determined by means of CAS assay calibrated with desferrioxamine B (DFOB). The siderophore was extracted from the supernatant with 200 g of XAD-2 resin. After absorption, the resin was washed thrice with 2 L of deionized water and the siderophore eluted with 100% methanol. The resulting eluate was evaporated to dryness and subsequently redissolved in ultrapure water. Further purification was achieved by means of a preparative XB-C18 reverse phase HPLC column (200 × 21.2 mm, 5 μm pore size, Kinetex by Phenomenex) with a flow of 8 mL/min and gradient elution from 30% 1 mM HCl to 60% acetonitrile (ACN) within 30 min. Peaks were detected at 210 nm. CAS-assay active fractions were pooled, evaporated to dryness, and stored at 4°C for later analysis.

### 2.6. Nuclear magnetic resonance spectroscopy

For the analysis via Nuclear magnetic resonance (NMR) spectroscopy, 25 mg of the substance of interest were dissolved in DMSO-*d*_6_ (Deutero, 99.96% D) and transferred into a 5 mm borosilicate tube. The spectra were obtained at 25°C on an Agilent DD2-600 NMR system, operating at 14.1 T with corresponding ^1^H, ^13^C, and ^15^N resonance frequencies of 599.8, 150.8, and 60.8 MHz, respectively, using a 5 mm oneNMR^™^ probe.

^1^H NMR spectra (conventional or using either Carr-Purcell-Meiboom-Gill (CPMG) pulse train or water signal suppression by pre-saturation, respectively) were recorded with an acquisition time of 1 s after applying a 2.8 μs (*π*/6) pulse, upon accumulation of 16 scans using 3 s relaxation delay. H,C-HSQC (heteronuclear single-quantum coherence) and H,C-HMBC (heteronuclear multiple-bond correlation) sequences applied gradient-selection and adiabatic pulses, acquiring 2 k × 512 and 2 k × 1 k complex points in *F*_2_ and *F*_1_, 48 transitions per *F*_1_ increment, with a relaxation delay of 1 s, respectively. For polarization transfer, (2 *J*)^−1^ delays of 4.0 and 62.5 ms were opted, corresponding to 125 Hz ^1^*J* in HSQC and 8 Hz *^n^J* in HMBC, respectively. H,N-HSQC and H,N-HMBC sequences applied gradient-selection and adiabatic pulses, acquiring 2 k × 256 and 2 k × 128 complex points in *F*_2_ and *F*_1_, 88 transitions per *F*_1_ increment, with a relaxation delay of 1 s, respectively. For polarization transfer, (2 *J*)^−1^ delays of 5.6 and 41.7 ms were opted, corresponding to 90 Hz ^1^*J* in HSQC and 12 Hz *^n^J* in HMBC, respectively. For homonuclear scalar and dipolar coupling detection, gradient-selected versions of ^1^H,^1^H-correlation spectroscopy (COSY) and rotating-frame Overhauser-enhancement spectroscopy (ROESY) experiments were performed upon acquisition of 2 k × 256 complex points in *F*_2_ and *F*_1_, 8 or 16 transitions per *F*_1_ increment, with relaxation delay of 1 s, respectively. ROESY mixing time was 200 ms. ^1^H and ^13^C as well as ^15^N chemical shift values are reported in ppm relative to internal TMS as well as external liquid ammonia, respectively.

### 2.7. Optimization of DFOE production by strain ATCH28^T^

The optimization of desferrioxamine E (DFOE) production by ATCH28^T^ was conducted in chemically defined M9 minimal medium (Geerlof, 2010), which was supplemented with 10% (w/v) NaCl. The addition of iron, as stated in the recipe, was omitted to ensure the ironlimitation necessary for siderophore production. Media parameters, that were investigated are concentration of NaCl (0–20% (w/v)), NH_4_Cl (0–0.3% (w/v)), LiCl (0–0.4 M), 0.02–1.5% (w/v) glucose, succinate or acetate, as well as addition of 2–8 mM threonine or lysine. The optimal pH value of the medium over the range from 7 to 8.5 was evaluated using 1 M NaOH for adjustments. Additionally, various combinations of glucose, NH_4_Cl and casamino acids as carbon and nitrogen sources were investigated to study their individual and cumulative effect on DFOE production. All experiments were carried out in 12-well plates, with 3 mL culture volume and examined after 7 days of incubation at 30°C. The concentration of DFOE was determined via analytical reverse phase XB-C18 HPLC column (250 × 4.6 mm, 5 μm pore size, Kinetex by Phenomenex), using a linear gradient from 5% 1 mM HCl to 60% ACN over the course of 50 min. As standard, commercially available DFOE (ASA Spezialenzyme) was facilitated.

## 3. Results and discussion

### 3.1. Genomic analysis

The amplification of the 16S rRNA genes of strains ATCHA^T^ and ATCH28^T^ yielded nearly complete sequences of 1,384 bp and 1,536 bp length, respectively, which have been deposited at GenBank under the accession numbers OM536009 (ATCHA^T^) and OM536011 (ATCH28^T^). On the basis of sequence similarity, the type species closest related to strain ATCHA^T^ were *Halomonas salicampi* (98.12%) and *Halomonas vilamensis* (98.05%). All other described members of the genus exhibited similarity scores below 98%. Strain ATCH28^T^ has closer relations to other members of *Halomonas*, with 9 type strains displaying sequence similarities higher than 98%. The four closest relatives found were *H. ventosae* (99.21%), *H. salina* (98.78%), *H. aestuarii* (98.7%), and *H. alimentaria* (98.39%). Those values are reflected in the inferred phylogenetic tree (Supplementary Figure S1), as strain ATCH28^T^ lies within a clade with *H. salina* and *H. ventosae*, while strain ATCHA^T^ is located within the same clade as *H. salicampi*.

**Figure 1:**
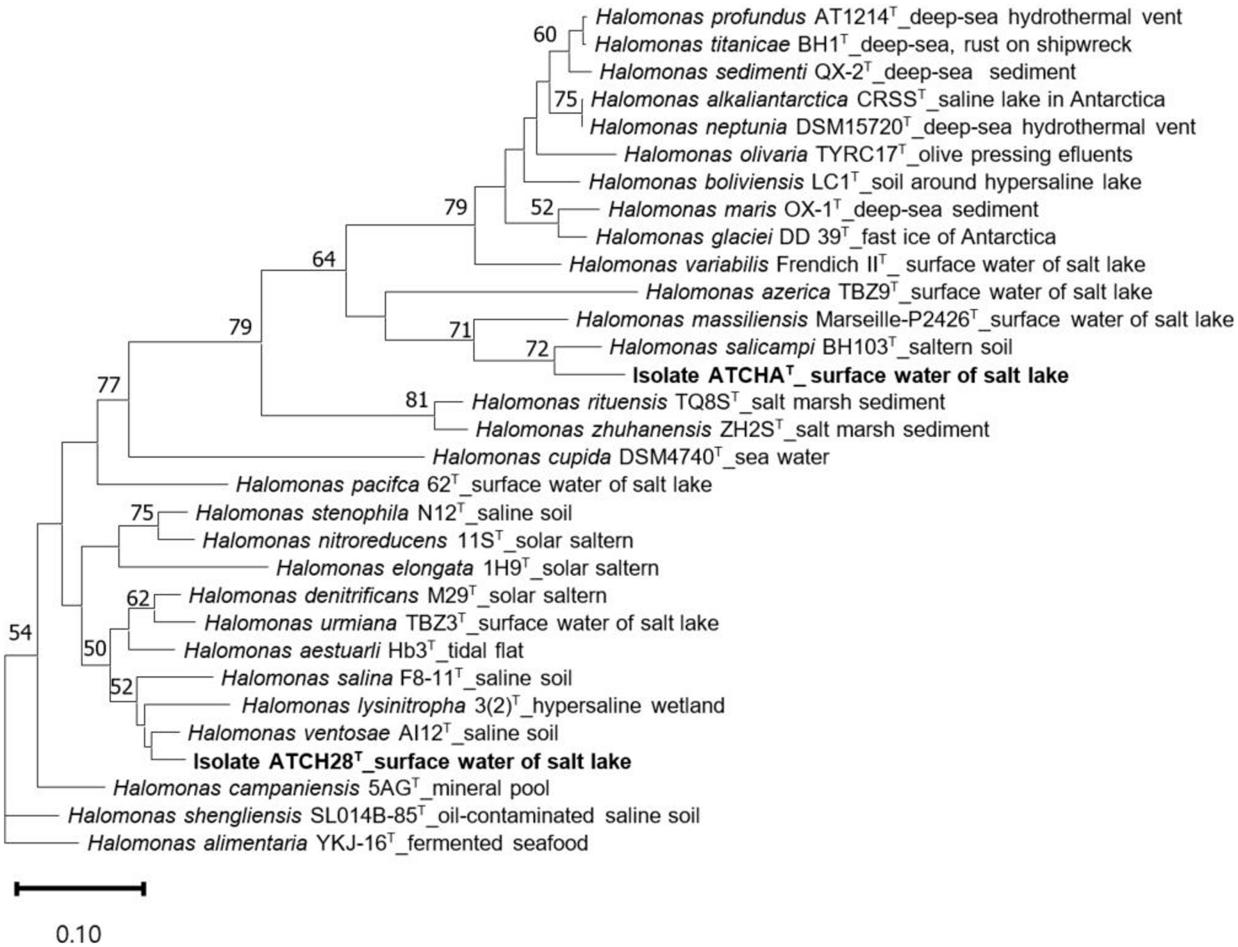
Maximum likelihood trees inferred from 81 single-copy core genes, demonstrating the relationship among ATCHA^T^, ATCH28^T^, and other type species within *Halomonas*. The source of isolation is shown at the branch tips. Only gene support indices greater than 50% are given at the branch points. The scale bar represents substitutions per nucleotide position.

The assembled draft genome of strain ATCHA^T^ is comprised of 176 contigs with a total length of 3,760,217 bp. Based on the presence and duplication of single-copy marker genes it has 100% completeness and 1.69% contamination. The sequence coverage is 59× and the N50 is 96,443 bp (Table 1). With 55.66 mol% the G + C content of the genome lies within the typical range of 51.4–74.3 mol% reported for the genus (Ventosa et al., 2021). The calculated ANI and dDDH values are lower than 88.3 and 35.7% (Table 2), respectively. The draft genome of strain ATCH28^T^ is 3,957,093 bp long and consists of 54 contigs with 100% completeness and 1.29% contamination. It has a sequence coverage of 119× and the sequence’s N50 is 452,947 bp (Table 1). The ANI and dDDH values between ATCH28^T^ and closely related species are <92.0% and <46.5% (Table 2), respectively. The genome has a G + C content of 66.52 mol%. Based on the analysis with ContEst16S there was no extra 16S rRNA gene sequence contamination present within both genomes. ANI and dDDH values calculated for both strains are below the generally accepted thresholds for the identification of new species of 96% (ANI) and 70% (dDDH) (Chun et al., 2018), indicating that they represent novel species within the genus *Halomonas*. The assembled genomes were deposited at NCBI under the accession numbers JAMJPJ000000000 (ATCHA^T^) and JAMJPK000000000 (ATCH28^T^). The phylogenomic tree (Figure 1) supports the core findings indicated by the phylogenetic tree, as strain ATCHA^T^ is located in a stable clade with *H. salicampi*, while strain ATCH28^T^ lies within the same clade as *H. ventosae* and *H. salina*. Comparing the isolation sources of all species depicted in the tree reveals, that the vast majority was isolated from hypersaline environments.

**Table 1:** Statistics of the genomes’ assemblies of strains ATCHA^T^ and ATCH28^T^.

Strain	Contigs	Total length [bp]	Coverage	N50	Completeness	Contamination
ATCHA^T^	176	3,760,217	59×	96,433	100%	1.69%
ATCH28^T^	54	3,957,093	119×	452,597	100%	1,29%

**Table 2:** ANI and dDDH values calculated between the genomes of strains ATCH28^T^ and ATCHA^T^ and the reference genomes of closely related species of *Halomonas.*

Reference genome	ATCHA^T^		ATCH28^T^	
	ANI [%]	DDH	ANI [%]	DDH [%]
*H. salina*	73.24	21.0	88.10	35.0
*H. ventosae*	73.44	20.2	92.03	46.5
*H. lysinitropha*	73.00	20.0	87.96	34.6
*H. aestuarii*	73.18	20.2	86.61	31.4
*H. alimentaria*	73.29	20.3	82.52	25.2
*H. salicampi*	88.33	35.7	73.90	20.2
*H. massiliensis*	81.20	23.8	74.90	21.0
*H. azerica*	74.82	20.4	73.55	20.2

**Figure 2:**
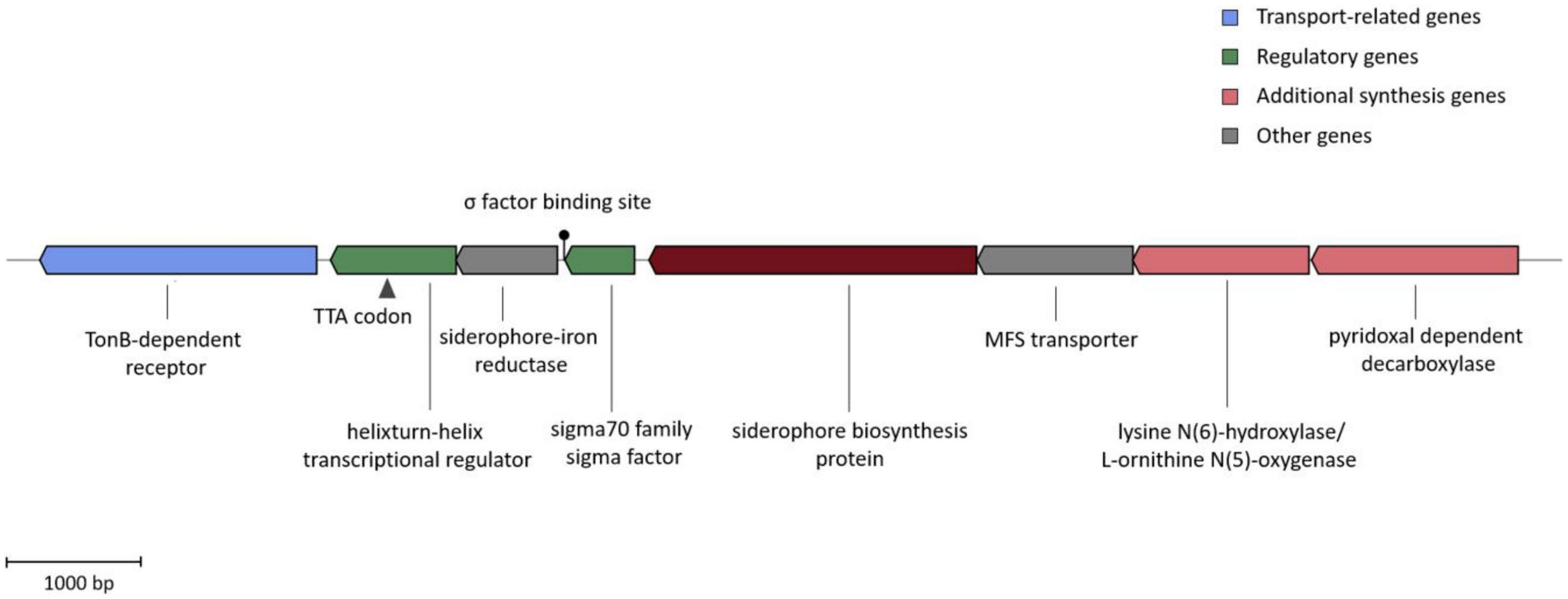
DFOE biosynthesis gene cluster present in strain ATCH28^T^. The color blocks represent different categories based on the AntiSMASH output. The TTA stop codon and Sigma factor binding site are indicated.

The analysis of ATCH28^T^’s genome via AntiSMASH indicates the presence of a non-ribosomal peptide synthetase (NRPS)-independent gene cluster facilitating siderophore biosynthesis. Based on the comparison with the MIBiG database (Terlouw et al., 2023) using AntiSMASH, the siderophore synthesized by strain ATCH28^T^ is very likely DFOE due to the high similarity score to the DFOE biosynthetic gene cluster present in *Pantoea agglomerans* (MIBiG accession BGC0001572). The cluster comprises the four genes *dfoACJS* and was found in various members of *Pantoea* and *Erwinia* (Smits and Duffy, 2011). The entire putative gene cluster identified in strain ATCH28^T^ consists of a TonB-dependent receptor, helix-turn-helix transcriptional regulator (with TTA stop codons), a siderophore-iron reductase, a sigma-70 family RNA polymerase sigma factor, a GCN5-related N-acetyltransferase, an MFS transporter, a lysine N(6)-hydroxylase/L-ornithine N(5)-oxygenase and a pyridoxal-dependant decarboxylase (Figure 2). In the biosynthesis of DFOE, the first enzyme involved is the pyridoxal dependent decarboxylase (DfoJ) converting L-lysine to cadaverine. Subsequently, utilizing O_2_, FAD and NADPH, the lysine N(6)-hydroxylase/L-ornithine N(5)-oxygenase (DfoA) might catalyze the oxygenation of terminal amino groups of cadaverine, yielding N-hydroxyl-cadaverine. The core enzyme of the cluster, the siderophore biosynthesis protein (DfoC), is predicted to have two domains, which act as acyl transferase and siderophore synthetase, respectively. In the first step, this enzyme is proposed to couple succinyl-CoA to N-hydroxyl-cadaverine, resulting in N-5-aminopentyl-N-(hydroxyl)-succinamic acid, which then is trimerized and cyclized to DFOE. The MFS transporter present in the cluster of strain ATCH28^T^ might correspond to DfoS, which is responsible for the cell export of DFOE (Smits and Duffy, 2011). Interestingly, as per the comparative analysis in antiSMASH performed via ClusterBlast module, the same structure of the entire gene cluster was identified in *H. cerina* CECT 7282, *Halomonas* sp. THAF5a, *Halomonas* sp. HG01, *Halomonas* sp. SL1, and *Halomonas* sp. THAF12. Furthermore, clusters exhibiting high similarities were found in *H. taeanensis* USBA-857 (87%) and *H. ventosae* CECT 5797 (85%; Supplementary Figure S2). This suggests, that the ability to produce DFOE might be more common within the genus *Halomonas* than previously reported.

**Figure 3:**
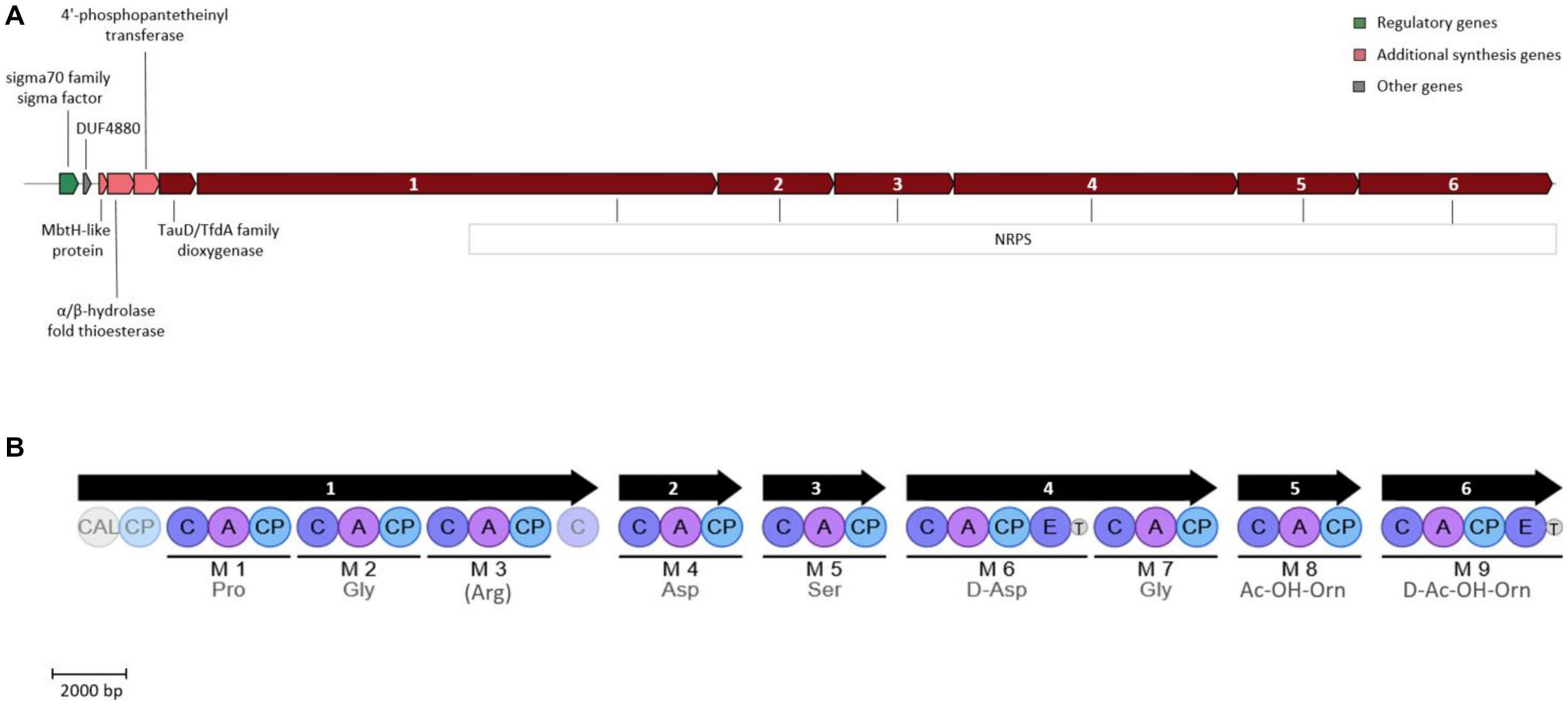
Siderophore biosynthetic gene cluster **(A)** and encoded NRPS modules **(B)** present in strain ATCHA^T^. The color blocks **(A)** represent different categories based on the AntiSMASH output. Numbers within NRPS genes indicate genes encoding the corresponding modules. Modules are indicated by black lines with predicted amino acids below. For amino acids in parenthesis, only 80% of the signature sequence matched the Stachelhaus code. Light shaded circles represent domains outside of modules. CAL, coenzyme A ligase domain; C, condensation domain; A, AMP binding domain; CP, peptidyl carrier protein domain; T, domain TIGR01720 of unknown function.

Opposed to strain ATCH28^T^, the siderophore produced by strain ATCHA^T^ is predicted to be NRPS-dependent. This type of synthesis is typical for the genus *Halomonas*, as it is commonly utilized in the production of acyl peptidic siderophores (Kem and Butler, 2015). Although the core structure of the siderophore produced by strain ATCHA^T^ could be predicted based on the putative biosynthetic gene cluster, it could not be conclusively identified using AntiSMASH. The gene cluster found in strain ATCHA^T^ shared the highest similarities to biosynthetic gene clusters responsible for the production of other siderophores, such as azotobactin D produced by *Azotobacter vinelandii* DJ (58% similarity), potashchelins A-D by *Halomonas* sp. MG34 (30% similarity), or crochelin A by *Azotobacter chroococcum* NCIMB 8003 (36% similarity). This might indicate, that the siderophore secreted by strain ATCHA^T^ has not yet been reported. The core biosynthetic genes of the cluster are indicated to be 7 genes encoding NRPS, as well as a TauD/TfdA family dioxygenase. Furthermore, it comprises a 4′-phosphopantetheinyl (4’-PP) transferase, an α/β-hydrolase fold thioesterase, an MbtH-like protein, a DUF4880 domain-containing protein, and a sigma 70 family sigma factor (Figure 3A). The construction of the siderophore is achieved via 9 modules encoded in the 7 NRPS genes. Generally, an NRPS module comprises an adenylation, condensation and a peptidyl carrier domain. As the substrate specificity of the adenylation domain is mediated by 10 amino acids lining the binding pockets, these signature sequences can be utilized to predict the amino acids that will be incorporated into the growing peptide chain (Stachelhaus et al., 1999). Figure 3B depicts the NRPS modules present in the biosynthetic gene cluster of strain ATCHA^T^, as well as the predicted core scaffold of the produced siderophore. In addition to the typical three domains, modules 6 and 9 contain epimerization domains, which convert the added L-amino acid into its D-enantiomer. A domain TIGR01720 is located directly after both epimerization domains. Although its function is yet unknown, its location indicates, that it is involved in the post-condensation modification of the peptide chain. MbtH-like protein-encoding genes often occur within NRPS gene clusters and are reported to play a role in promoting the folding, stability and activity of the NRPS enzymes (Zwahlen et al., 2019). The 4’-PP transferase likely is needed for the activation of the peptidyl carrier protein by transferring a 4’PP cofactor derived from coenzyme A to the enzyme’s conserved serine residue in order to convert it to its holo form (Miller and Gulick, 2016). The TauD/TfdA family dioxygenase encoded in the cluster might hydroxylate the aspartate residues forming α-hydroxycarboxylic acid moieties, which are typical functional groups in the chelation of metals by siderophores. The α/β-hydrolase fold thioesterase gene present in the cluster might play a role in the termination of the siderophore biosynthesis, which is commonly achieved via intramolecular cyclization or hydrolysis of the peptide chain (Kem and Butler, 2015). A comparison of the putative core amino acid sequence of the siderophore produced by strain ATCHA^T^ with those commonly occurring within the genus *Halomonas* further supports the novelty of the siderophore, as none of the reported sequences matches the one of the herein investigated compound (Supplementary Figure S3).

### 3.2. Phenotypic characteristics

Cells of strain ATCH28^T^ and ATCHA^T^ are short, gram-stain-negative, rods, which are motile by means of monotrichous or lophotrichous flagella, respectively. Anaerobic growth was not observed. The colonies of both strains are round, convex and cream-colored. They are mainly neutrophilic, growing from pH 6–9.5 with optima at pH 7.5 (ATCHA^T^) or 8.5 (ATCH28^T^). The strains were found to be moderately halophilic, with optimal growth at 10% (w/v) NaCl content and growth ranges from 3–20 or 0.5–17% (w/v). Both strains could grow within the temperature range of 4–40°C, while optimal growth was achieved at 30°C. Nitrate reduction was found in strain ATCH28^T^, but not ATCHA^T^. They are not able to hydrolyze gelatin, starch, DNA or casein, but could hydrolyze aesculin. Hydrogen sulfide was not produced by these strains. Distinct phenotypic characteristics that differentiate strains ATCHA^T^ and ATCH28^T^ from closely related type species of the genus *Halomonas* are the utilization of specific carbon sources, their ability to hydrolyze aesculin, NaCl range and optima, as well as the inability of strain ATCHA^T^ to reduce nitrate. Further differentiating properties of both strains are listed in Table 3.

**Table 3:** Phenotypic characteristics that distinguish between strain ATCHA^T^, ATCH28^T^, and type strains of closely related species of the genus *Halomonas*.

Characteristic	ATCHA^T^	ATCH28^T^	*H. salicampi* ^a^	*H. vilamensis* ^b^	*H. ventosae* ^c^	*H. salina* ^d^
Cell shape	Rod	Short rod	Rod	Rod	Short rod	Short rod
Cell size (μm)	3.6–6.0 × 0.8	2.0–3.0 × 1.0	1.1–1.3 × 0.3–0.5	2.0–4.0 × 1.0–1.6	1.2–1.5 × 0.7–0.8	2.0–2.5 × 0.7–0.8
Pigmentation	Cream	Cream	Cream	Cream-pink	Cream	yellowish-cream
Flagellum	Lopho	Mono	Lopho	Lopho	ND	Nonmotile
NaCl range (% w/v)	3–20	0.5–17	0–23	1–25	3–15	2.5–20
NaCl optimum (% w/v)	10	10	14	5–10	6–9	5
Temperature range (°C)	4–40	4–40	10–55	5–40	15–50	15–40
Temperature optimum (°C)	30	30	28	30	ND	32
pH range	6.0–9.5	6.0–9.0	7.0–10.8	5.0–10.0	6.0–10.0	6.0–10.0
pH optimum	7.5	8.5	8.5	7.0–8.0	ND	7.2
DNA G + C content (mol%)	55.7	66.5	54.7	55.0	72.6–74·6	60.7–64.2
Nitrate reduction	−	+	+	+	+	+
Production of H_2_S	−	−	−	−	+	−
Hydrolysis of:						
Aesculin	+	+	−	−	−	−
Gelatin	−	−	−	+	−	−
DNA	−	−	−	−	−	−
Utilization of:						
Glycerol	−	−	+	−	+	ND
D-Galactose	−	+	−	−	+	−
D-Glucose	−	+	+	−	+	−
D-Mannose	−	−	−	−	ND	+
D-Sorbitol	−	−	−	−	+	+
D-Trehalose	−	+	+	−	ND	+
Maltose	−	+	+	−	+	+
Sucrose	−	+	+	−	ND	+
Turanose	−	+	−	−	ND	ND
Acetic acid	+	+	ND	+	ND	+
Citric acid	+	−	−	−	+	+
Lactic acid	−	−	ND	−	+	+
Malic acid	−	+	ND	+	+	+
Propionic acid	+	+	ND	−	+	−
Gluconic acid	−	+	−	−	+	+
L-Alanine	+	+	ND	+	−	+
L-Serine	−	+	ND	−	ND	−

+, positive; −, negative; ND, no data available.

a Lee et al. (2015).

b Menes et al. (2011).

c José Martínez-Cá et al. (2004).

d Valderrama et al. (1991).

### 3.3. Chemotaxonomic characteristics

Grown in the isolation medium, the major fatty acids of strain ATCHA^T^ are 19:0 cyclo (40.08%), 16:0 (23.15%), and 17:0 cyclo (20.74%), while the main fatty acids of ATCH28^T^ are 18:1 cyclo ω7c (27.48%), 16:0 cyclo ω7c (18.84%), 16:0 (18.64%), 19:0 cyclo (14.82%). Table 4 lists all fatty acids found in amounts of more than 1%. In accordance with literature, the main respiratory quinone identified in both strains was Q9.

**Table 4:** Cellular fatty-acid composition (%) of strains ATCHA^T^ and ATCH28^T^ grown in the respective isolation media; −, negative or less than 1%.

Fatty acid	ATCHA^T^	ATCH28^T^
C_10:0_	2.13	4.06
C_10:0_ 3-OH	–	1.03
C_12:0_ 3-OH	5.61	8.83
C_16:0_	23.15	18.64
C_16:1_ ω7c	5.61	18.81
C_17:0_ cyclo	20.74	4.9
C_18:1_ ω7c	–	27.48
C_19:0_ cyclo	40.08	14.82

### 3.4. Production and purification of siderophores

Based on the results of the CAS liquid assay, the culture supernatant of ATCHA^T^ contained 15 μM siderophores. Although extraction of the compound was possible with XAD2, a substantial loss of the substance was observed, as only a volume of 10 mL with a concentration of 120 μM could be obtained from 6 L culture volume. The purification via HPLC was expected to result in further loss of the compound. While the presence of siderophores is clearly indicated by the positive reaction of the CAS liquid assay and genomic analysis, the amounts produced were too low for the exhaustive structural characterization of the compound. As this study aimed to find organisms capable of high-yield production, further investigation of the siderophore produced by strain ATCHA^T^ was deemed to not lie within the scope of our investigations.

Opposed to this, the culture supernatant of strain ATCH28^T^ contained 85 μM of siderophore after 7 days of cultivation. Extraction of the siderophore via XAD2 yielded 110 mL of a slightly orange liquid with a concentration of 840 μM. During HPLC purification, a single CAS active peak was collected from 17.25 to 17.5 min. The evaporation of the collected fraction yielded 40 mg purified siderophore.

### 3.5. Nuclear magnetic resonance spectroscopy

**Figure 4:**
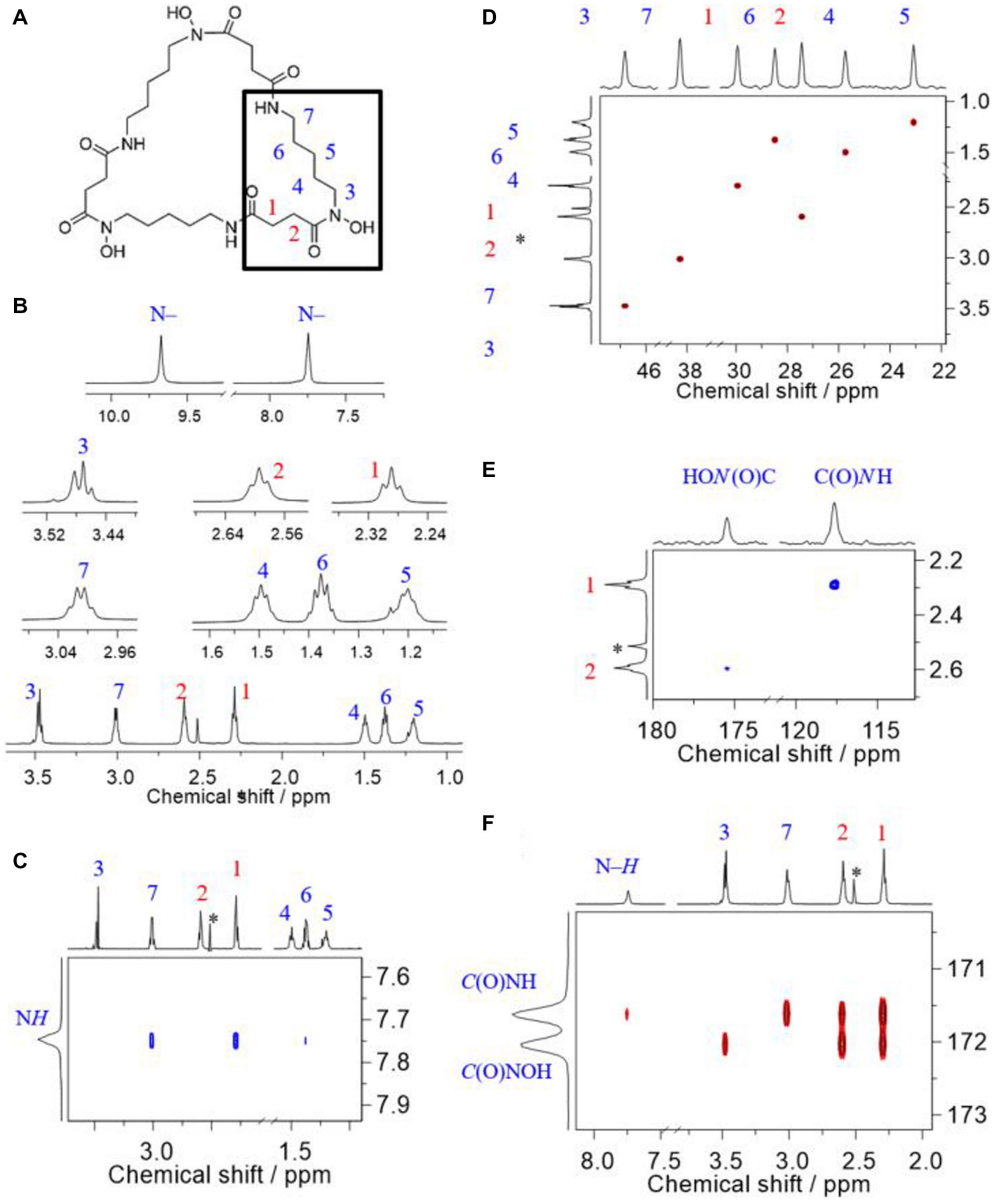
Comprehensive ^1^H, ^13^C, and ^15^N NMR signal assignment to corresponding sites in desferrioxamine E, recorded at 14.1 T in DMSO-*d*_6_ at 25°C. **(A)** Generic structure together with atomic labeling, **(B)** ^1^H NMR of methylene groups (bottom) together with magnifications of all signals, **(C)** Trace of the H, H-ROESY showing the spatial correlations associated with the amide proton, **(D)** H,C-HSQC signals of methylene groups, **(E)** H,N-HMBC signals associated with the nitrogen carbon atoms, and **(F)** H,C-HMBC associated with the carbonyl carbon atoms in the amide and hydroxamate groups. The asterisk denotes residual signal of the solvent; chemical shift values reported in ppm relative to TMS.

The siderophore produced by strain ATCH28^T^ was unambiguously identified as DFOE. The following considerations and the NMR signal assignment are according to the labeling along with the generic structure depicted in Figure 4A.

Based on the given structure of DFOE, the expected and the observed number of NMR signals matches; that is, seven ^1^H, nine ^13^C, and two ^15^N signals, respectively. This number of signals is due to the building block, *N*-hydroxy-*N*-succinyl cadaverine (HSC; Fujita and Sakai, 2013), three molecules of which form DFOE (see highlighted residue in Figure 4A), featuring a molecule with threefold symmetry. Two isolated ^1^H spin systems (cf. Supplementary Figure S4), respectively corresponding to the succinyl and the cadaverine residues, allow for easy recognition of HSC as the only building block. That is, the succinyl component comprises two methylene groups, 1 and 2, as well as the two carbonyl carbons, and the *N*-hydroxy cadaverine residue consists of five methylene groups, 3 through 7, and the two nitrogen atoms. In DFOE, the latter together with the succinyl carbonyl carbons form the hydroxamate and amide groups, respectively. Any modification such as an additional functional group would alter the characteristic pattern of the coupling spins. In combination with molecular weight data from mass spectrometry, the only possible molecule consequently is DFOE. Linear molecules such as bisucaberin B or DFOB can be ruled out owing to both the reduced symmetry as well as the molecule’s terminating amino and methyl groups, respectively, which are associated with a higher number and different appearance of the signals.

To the best of our knowledge, the first report on ^1^H and ^13^C NMR spectra of DFOE, also referred to as nocardamine, is by Maehr et al. (1977), serving as authoritative reference for comparison. Later reports, such as by Yamanaka et al. (2005) or Spasojević et al. (1999), among other methods, employ NMR spectroscopy to verify the identity of their DFOE obtained from *Streptomyces* spp.

One of the most recent reports is by Acquah et al., who obtained their NMR spectra under comparable conditions as we did (e.g., field strength and solvent). Our spectra (Figures 4A–F) are in excellent agreement with those of Acquah et al. (2020). The authors provided signal assignment along with their spectra; however, although acquired in DMSO-*d*_6_, they do not show the ^1^H signals associated with the N–H and N–OH groups (cf. Figure 4B). Based on our 2D NMR correlation experiments we agree with their assignment except for the ^13^C signals of the two carbonyl carbons of the amide and hydroxamate groups, respectively. That is, we are convinced that the hydroxamate carbonyl carbon is the one less shielded instead of the amide carbonyl carbon.

Since the attribution of the C and N atoms in either of these functional groups builds upon their respective heteronuclear multiple-bond correlation (HMBC) to ^1^H nuclei, correct assignment of the latter is essential, particularly for those hydrogens adjacent to the functional groups, viz. H’s 1, 2, 3, and 7.

The methylene ^1^H signals most downfield (*δ*_H_ 3.47 and 3.01 ppm) are attributed to sites adjacent to the nitrogen atoms. Simply by dint of the appearance as a triplet and a quartet-like signal, attribution of these signals to the corresponding sites 3 and 7, respectively, is possible (Figure 4B). The close proximity of H7 and H1 but not H3 and H2 as seen from the ROESY (Figure 4C) corroborates that the first two methylene groups neighbor the amide group. From the ^1^H,^15^N-HMBC spectrum opted for 8 Hz, thus emphasizing coupling via three bonds (^3^*J*), the methylene groups 1 and 2 can be assigned. The ^15^N nuclei in the C(O)NH and C(O)NOH groups, respectively, resonate at about *δ*_N_ 118 and 175 ppm (Figure 4E). The ^3^*J*_N,H_ render the assignment of 1 and 2 unambiguous, as the amide nitrogen and H1 as well as the hydroxamate nitrogen and H2 are separated by three bonds, respectively. The coupling of the latter pair is quite weak (due to site exchange with water, see Supplementary Figure S5) but visible. Finally, upon correlation between the ^1^H of the amide group as well as methylene groups 1, 2, 3, and 7, the carbonyl carbon signals can be identified (Figure 4F). In this correlation spectrum the ^2^*J*_C,H_ and ^3^*J*_C,H_ are emphasized. Therefore, 1 and 2 show correlations to either carbonyl carbon, which is not helpful for assignment. But 3 and 7, respectively, show one correlation only, namely ^3^*J* between H3 and the hydroxamate carbon, and ^3^*J* between H7 and the amide carbon. Additionally, the amide proton shows ^2^*J*_H,C_ to the amide carbonyl carbon. Consequently, the latter attributes to the signal of smaller ^13^C chemical shift.

### 3.6. Optimization of DFOE production by strain ATCH28^T^

As the biosynthesis of DFOE is untypical within the genus and strain ATCH28^T^ produced unusually high amounts of siderophore, the effect of various media components was investigated with the aim of further increasing the production and gaining a deeper understanding of the parameters that influence the secretion of the compound.

**Figure 5:**
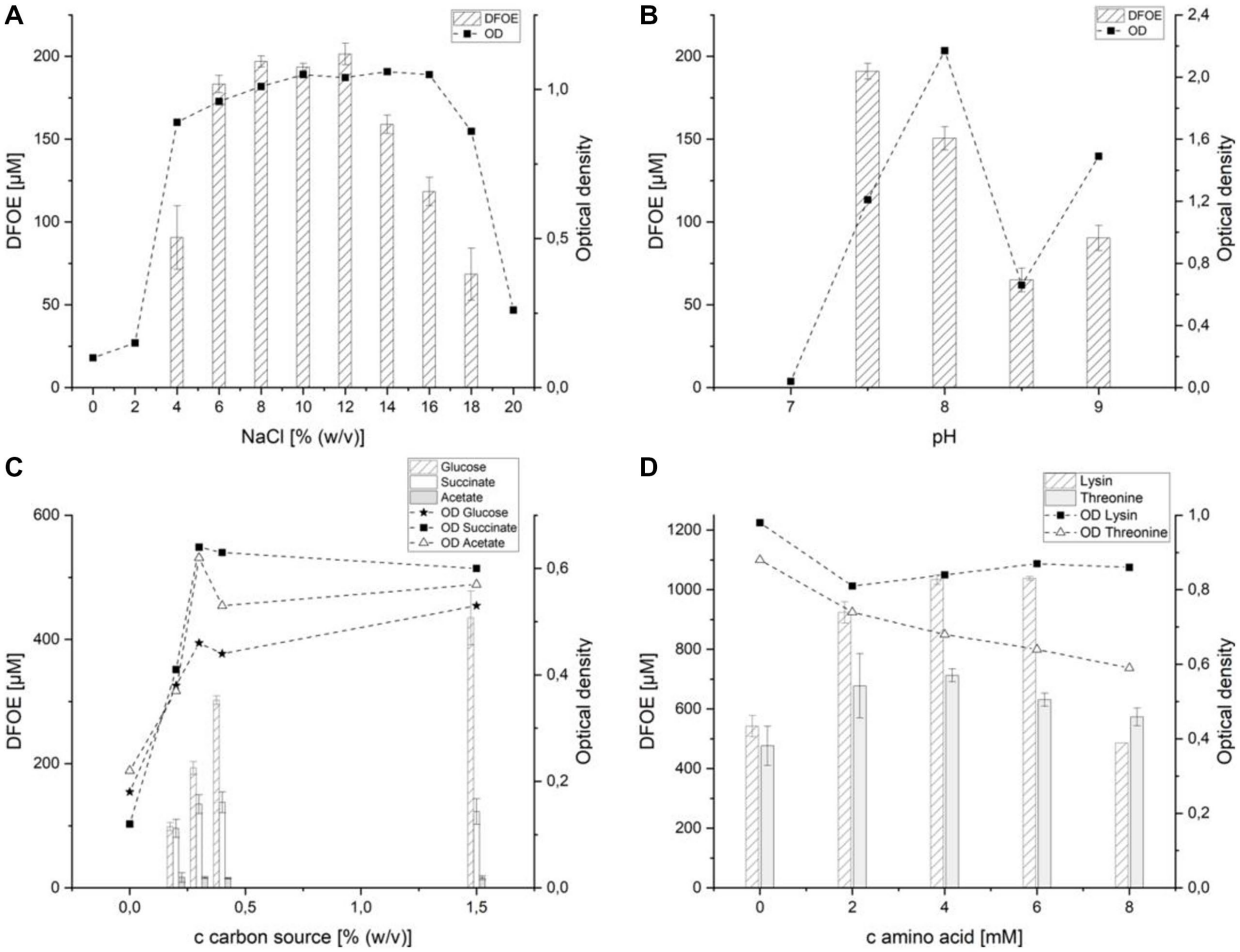
Effect of various media components on the production of desferrioxamine E by strain ATCH28^T^. **(A)** sodium chloride, **(B)** pH-value, **(C)** carbon source, **(D)** lysine and threonine.

The member of *Halomonas* described herein is a halophilic organism, which is why the salt content of the medium was expected to indirectly influence the siderophore secretion by affecting the amount of biomass produced. In M9 containing 0–2% (w/v) NaCl, growth of the organism was negligible and no siderophore production could be observed. Although the OD measured after 7 days of incubation was similar within the range of 4–16% (w/v) NaCl, the DFOE secretion followed a different trend. While 91 (±14) μM of the siderophore was detected at 4% (w/v) NaCl, an increase to 6% (w/v) NaCl resulted in 183 (±4) μM DFOE measured in the supernatant. Starting at 14% (w/v) NaCl, the secretion of DFOE by strain ATCH28^T^ decreased and halted completely at 20% (w/v) NaCl. At the same time growth was reduced at NaCl contents above 16% (w/v) and only little growth could be observed at 20% (w/v) NaCl content. The optimal amount of NaCl was found to be in the range of 8–12% (w/v) NaCl, reaching concentrations of up to 201 (±5) μM DFOE in the culture supernatant (Figure 5A). This suggests that other than expected, NaCl plays a direct role in the DFOE production by strain ATCH28^T^. Similar trends have been found in other siderophore-producing species, such as *Shewanella putrefaciens* (Soe et al., 2012) or *Bacillus subtilis* (Hoffmann et al., 2002). It was reported, that osmotic stress had an effect on the induction of genes involved in the iron uptake, similar to the one caused by iron-depleted conditions (Hoffmann et al., 2002). For further experiments, the media were supplemented with 10% (w/v) NaCl.

Because the strain originates from an environment that contains 6 mM lithium, the effect of its salt on siderophore production was investigated (Supplementary Figure S6A). Supplementation of M9 with 300 mM LiCl led to an increase in DFOE production by 28 (±4) μM. The addition of more LiCl did not affect growth or siderophore production to a greater extent. This suggests that lithium chloride influenced the production of DFOE, however, its effect was comparatively low.

The effect of the pH value of the medium on DFOE production was observed from pH 7 to 8.5 (Figure 5B). At pH 7 no substantial growth was observed and as a result, no siderophore was produced. At the optimum of pH 7.5 DFOE concentrations of 191(±5) μM were detected, which decreased to 150 (±7) μM at pH 8. A further increase in pH value led to a reduction in the biosynthesis of DFOE. The production of DFOE followed the same trend as the optical density (data not shown), which suggests that the pH value only plays an indirect role in the siderophore secretion.

Testing various combinations of glucose, NH_4_Cl and casamino acids clearly showed the significance of ammonium and glucose in the production of DFOE (Supplementary Figure S6B). While the highest optical density was achieved in M9 containing glucose and casamino acids, no siderophore was found in the supernatant. Opposed to the growth in IM1, the strain was also not able to efficiently utilize casamino acids as the sole carbon source. Good growth and production of 153 (±6) μM DFOE could be achieved in M9 containing both glucose and NH_4_Cl. Although the same optical density was measured in the culture containing all three components, the siderophore concentration was considerably higher, reaching 259 (±5) μM. This might be attributed to the strain utilizing the amino acids contained in the casamino acids as building blocks in the synthesis of DFOE.

Because NH_4_Cl was indicated to be integral to the induction of DFOE biosynthesis, the effect of an increase in its concentration on the production of DFOE was investigated. Although small amounts of ammonium are indicated to be indispensable for the production of the siderophore, increasing the concentration of NH_4_Cl in the medium above 0.05% did not significantly affect the production (Supplementary Figure S6C).

The effect of various carbon sources on DFOE production is depicted in Figure 5C. Succinate was chosen, as it represents a derivative of succinyl-CoA, which is needed by DfoC in the biosynthesis of DFOE. Similarly, acetate, a derivative of acetyl-CoA, was included, as it is a component in the synthesis of DFOB (Giddings et al., 2021). As expected, acetate as the sole carbon source did not positively influence siderophore production. Each concentration tested above 0% (w/v) yielded roughly 16 μM DFOE. Moreover, DFOB production was not observed, which suggests the tight regulation of the flux of acetate and succinate in the cellular pool. Utilizing succinate as sole carbon source, the highest concentration of DFOE produced by strain ATCH28^T^ was approximately 135 μM at 0.3 and 0.4% (w/v). This indicates that the addition of derivatives of succinyl-CoA does not substantially increase the biosynthesis of DFOE by strain ATCH28^T^. The highest amount of siderophore was produced in the medium containing glucose as sole carbon source. Here, an increase in concentration of the carbon source led to a strong increase in DFOE production. While only 98 (±5) μM were produced in the medium containing the original amount of 0.2% (w/v) glucose, 435 (±31) μM DFOE could be achieved by using 1.5% (w/v) of the carbon source. The higher production rate of DFOE achieved by utilizing glucose might be based on the necessity of NADPH for the reaction catalyzed by DfoA. The coenzyme can be generated in the Entner–Doudoroff pathway and pentose phosphate pathway, both of which require glucose-6-phosphate (Spaans et al., 2015). Hence, greater amounts of glucose may result in a higher availability of NADPH.

As casamino acids had a positive effect on the production of DFOE, the supplementation of the medium with lysine or threonine was tested. Lysine was chosen, as it represents the initial substrate in the biosynthesis of DFOE. The addition of lysine resulted in a 200% increase in DFOE present in the culture supernatant, reaching a maximum of 1,038 (±5) μM at 6 mM lysine (Figure 5D). Threonine was expected to increase the siderophore production of strain ATCH28^T^, as it was proven to positively influence the production of desferrioxamine B by *Streptomyces pilosus* (Chiani et al., 2010). As anticipated, the supplementation of the medium with threonine led to an increase in siderophore production. The amount of DFOE in the culture supernatant rose from 477 (±46) μM to 713 (±15) μM DFOE with the addition of 4 mM of the amino acid.

Compared to other siderophore-producing species of the genus *Halomonas*, which synthesized 40 μM sodachelin (Serrano Figueroa et al., 2015) or 25.4 μM halochelin (Matsui and Nishino, 2016), respectively, significantly higher concentrations of 85 μM were obtained with strain ATCH28^T^ in IM1. This concentration could be substantially increased to more than 1 mM or roughly 600 mg/L, by utilizing M9 with adjustments in NaCl content and pH-value as well as the addition of the DFOE precursor Lysine. Although this concentration is not yet on par with the ones reported for *Streptomyces parvulus* (2 g/L; Gáll et al., 2016) or *Streptomyces olivaceus* (14 g/L; Meiwes et al., 1990), first small-scale optimizations made within this work indicate, that strain ATCH28^T^ might be a viable organism for the industrial production of the siderophore. Further optimizations of the production medium, as well as the culture strategy, such as the utilization of fed fermentation, could result in even higher concentrations of DFOE produced. Especially industries in arid countries might profit from this, as production in seawater might be a feasible option.

### 3.7. Taxonomic conclusions

The 16S rRNA gene sequences’ similarity scores, differences in G + C contents, dDDH and ANI values, as well phenotypic and chemotaxonomic characteristics of the strains described herein clearly prove that ATCHA^T^ and ATCH28^T^ represent two novel species of the genus *Halomonas* for which we propose the designations *Halomonas llamarensis* sp. nov. and *Halomonas gemina* sp. nov., respectively.

### 3.8. Description of *Halomonas llamarensis* sp. nov.

*Halomonas llamarensis* sp. nov. (lla.mar.en’sis. N.L. fem. Adj. *llamarensis,* referring to Salar de Llamará, Chile).

Cells are strictly aerobic living rods (3.6–6 μm × 0.8 μm) and motile by means of lophotrichous flagella. Cultured on IM4 at 30°C for 6 days, colonies are cream, translucent, circular, convex and smooth with clearly defined margins and a diameter of 2–3 mm. Growth occurs between 4–40°C (optimum 30°C), 3–20% (w/v) NaCl (optimum 10%) and pH 6–9 (optimum 7.5). The strain is not capable to grow in the absence of NaCl. It is positive for oxidase as well as catalase activities and negative for H_2_S Production. The cells are not able to hydrolyze starch, casein, and DNA. Growth was observed with L-alanine, L-glutamic acid, N-acetyl-D-glucosamine, citric acid, α-keto-glutaric acid, propionic acid and acetic acid as sole carbon source. No growth was observed on dextrin, D-maltose, D-trehalose, D-cellobiose, D-gentiobiose, sucrose, D-turanose, stachyose, D-raffinose, α-D-lactose, D-melibiose, B-methyl-D-glucoside, D-salcin, N-acetyl-β-D-mannosamine, N-acetyl-D-galatosamine, N-acetyl-neuraminic acid, α-D-glucose, D-mannose, D-fructose, D-galactose, 3-methyl glucose, D-fucose, L-fucose, L-rhamnose, inosine, D-serine, D-sorbitol, D-mannitol, D-arabitol, myo-inositol, glycerol, D-glucose6-PO_4_, D-fructose6-PO_4_, D-aspartic acid, glycyl-L-proline, L-arginine, L-aspartic acid, L-histidine, L-pryoglutamic acid, L-serine, D-galacturonic acid, L-galactonic acid lactone, D-gluconic acid, D-glucoronic acid, glucuronamide, mucic acid, quinic acid, D-saccharic acid, p-hydroxy-phenylacetic acid, methyl pyruvate, D-lactic acid methyl ester, L-lactic acid, D-malic acid, L-malic acid, bromo-succinic acid, γ-amino-butyric acid, α-hydroxy-butyric acid, β-hydroxy-D,L-butyric acid, α-keto-butyric acid, acetoacetic acid or formic acid. The strain is resistant to vancomycin and fusidic acid but susceptible to troleandomycin, rifamycin, minocycline, nalidixic acid and aztreonam. In API 20NE test kit, the cells are positive for hydrolysis of esculin, but negative for indole production, nitrate reduction and hydrolysis of gelatine, 4-nitrophenyl-β-D-glucopyranoside (PNPG), urea and L-arginine. Activities of alkaline phosphatase, esterase, esterase lipase, valine arylamidase, acid phosphatase and naphthol-AS-BI-phosphohydrolase were observed. Additionally, ATCHA^T^ is able to biosynthesize siderophores in an NRPS-dependent manner. Main respiratory quinone is ubiquinone 9. Predominant fatty acids are 19:0 cyclo, 16:0 and 17:0 cyclo.

The type strain is ATCHA^T^ (=DSM 114476 = LMG 32709) and was isolated from the surface water of Salar de Llamará in northern Chile. The genomic G + C content of the type strain is 55.7 mol%. The GenBank accession numbers for the type strain’s 16S rRNA sequence and draft genome are OM536009 and JAMJPJ000000000, respectively.

### 3.9. Description of *Halomonas gemina* sp. nov.

*Halomonas gemina* (ge.mi.na L. fem. Adj. *gemina*, twin, double).

Cells are short rods (2.0–3.0 μm × 1 μm) and typically present in pairs. They are strictly aerobic and motile by means of a monotrichous flagellum. On IM1, colonies are cream, smooth, opaque, and circular with clearly defined margins and diameters of 1–4 mm. Growth was observed at 4–40°C (optimum 30°C), 3–12% NaCl (optimum 7%), and pH 6–10 (optimum 8.5). No growth in the absence of NaCl and positive for catalase and oxidase. The cells are negative for the production of H_2_S and hydrolysis of casein. Starch, DNA and gelatin. The strain is able to utilize D-maltose, D-trehalose, Sucrose, D-turanose, α-D-glucose, D-galactose, Inosine, D-serine, glycyl-L-proline, L-alanine, L-glutamic acid, L-serine, D-gluconic acid, D-malic acid, L-malic acid, β-hydroxy-D, L-butyric acid, propionic acid and acetic acid as sole carbon sources. No growth was observed with dextrin, D-cellobiose, D-gentiobiose, stachyose, D-raffinose, α-D-lactose, D-melibiose, B-methyl-D-glucoside, D-salcin, N-acetyl-D-glucosamine, N-acetyl-β-D-mannosamine,N-acetyl-D-galatosamine, N-acetyl-neraminic acid, D-mannose, D-fructose, 3-methyl glucose, D-fucose, L-fucose, L-rhamnose, D-sorbitol, D-mannitol, D-arabitol, myo-inositol, glycerol, D-glucose-6-PO_4_, D-fructose-6-PO_4_, D-aspartic acid, L-arginine, L-aspartic acid, L-glutamic acid, L-histidine, L-pyroglutamic acid, D-galacturonic acid, L-galactonic acid lactone, D-gluconic acid, D-glucuronic acid, glucuronamide, mucic acid, quinic acid, D-saccharic acid, p-hyroxy-phenylacetic acid, methyl pyruvate, D-lactic acid methyl ester, L-lactic acid, citric acid, α-keto-glutaric acid, bromo-succinic acid, γ-amino-butyric acid, α-hydroxy-butyric acid, α-keto-butyric acid, acetoacetic acid or formic acid as sole carbon source. Cells are susceptible to fusidic acid, troleandomycin and minocycline but resistant to aztreonam, nalidixic acid, vancomycin, lincomycin and rifamycin. With API 20NE test kit, they tested positive for reduction of nitrate, hydrolysis of esculin and indole production, but negative for hydrolysis of urea and L-arginine. Activities for alkaline phosphatase, esterase lipase, leucine arylamidase, valine arylamidase, trypsin, naphthol-AS-BI-phosphohydrolase, β-galactosidase and α-glucosidase were observed. ATCH28^T^ has the ability to produce the siderophore desferrioxamine E. Main respiratory quinone is ubiquinone 9. Predominant fatty acids are 18:1 cyclo ω7c, 16:0 cyclo ω7c, 16:0, 19:0 cyclo.

The type strain is ATCH28^T^ (=DSM 114418 = LMG 32708) and was isolated from Laguna Leija in northern Chile. The genomic G + C content is 66.5 mol%. The GenBank accession numbers of the 16S rRNA sequence and the draft genome are OM536011 and JAMJPK000000000, respectively.

## Data availability statement

The datasets presented in this study can be found in online repositories. The names of the repository/repositories and accession number(s) can be found in the article/Supplementary material.

## Author contributions

CH, SS, JK, SW, KK, and FL performed the experiments. LR provided the environmental samples. KP, RJ, SK, and VS supervised the study. RJ revised the manuscript. All authors contributed to the article and approved the submitted version.

## Funding

This study was conducted as part of the SIDEREC project, supported by the ERA-MIN 2 call (German grant number 033RU01 0A and Chilean grant number Nº51-ANID).

## Conflict of interest

The authors declare that the research was conducted in the absence of any commercial or financial relationships that could be construed as a potential conflict of interest.

## Publisher’s note

All claims expressed in this article are solely those of the authors and do not necessarily represent those of their affiliated organizations, or those of the publisher, the editors and the reviewers. Any product that may be evaluated in this article, or claim that may be made by its manufacturer, is not guaranteed or endorsed by the publisher.

## Supplementary material

The Supplementary material for this article can be found online at: https://www.frontiersin.org/articles/10.3389/fmicb.2023.1194916/full#supplementary-material

## Footnotes

^1^ https://lpsn.dsmz.de/family/halomonadaceae

^2^ https://lpsn.dsmz.de/genus/halomonas

^3^ http://leb.snu.ac.kr/ubcg2/genes
