# Supplementary material for: Halomonas gemina sp. nov. and Halomonas llamarensis sp. nov., two siderophore-producing organisms isolated from high-altitude salars of the Atacama Desert
Source: Front Microbiol. 2023 Jun 12;14:1194916. doi: 10.3389/fmicb.2023.1194916 (PMC10291192; doi:10.3389/fmicb.2023.1194916)
Supplement: Supplementary file 1 [file Data_Sheet_1.pdf]

Supplementary Material

Halomonas gemina sp. nov. and Halomonas llamarensis sp. nov., two siderophore-producing organisms isolated from high-altitude salars of the Atacama Desert

Christian Hintersatz, Shalini Singh, Luis Antonio Rojas, Jerome Kretzschmar, Sean Ting-Shyang Wei, Khushal Khambati, Sabine Kutschke, Falk Lehmann, Vijai Singh, Rohan Jain*, Katrin Pollmann

*** Correspondence:** Rohan Jain, R.Jain@hzdr.de

1 Supplementary Figures

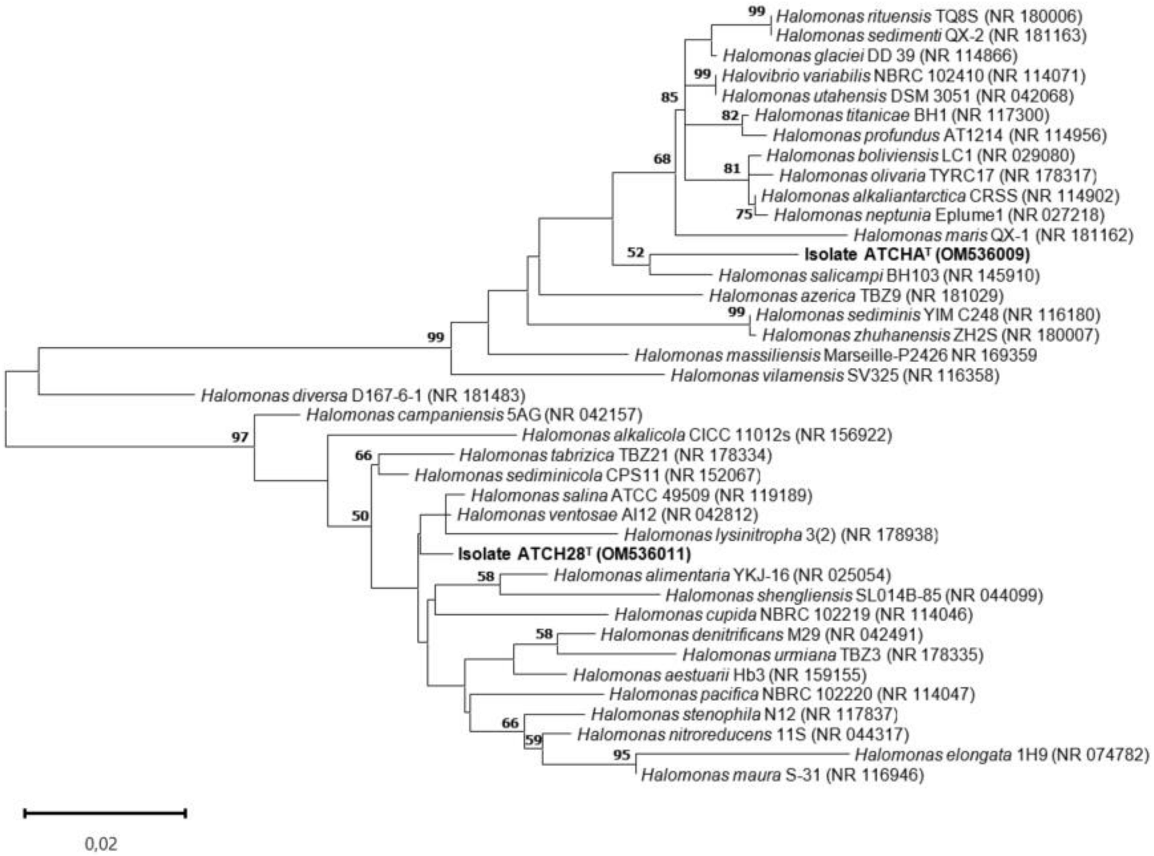

Figure S1. Maximum likelihood tree inferred based on 16S rRNA gene sequences showing the relationgship among ATCHA^T, ATCH28^T and closely related type species within *Halomonas*. Only bootstrap values greater than 50% are given at the branch points. The scale bar represents subsitutions per nucleotide position.

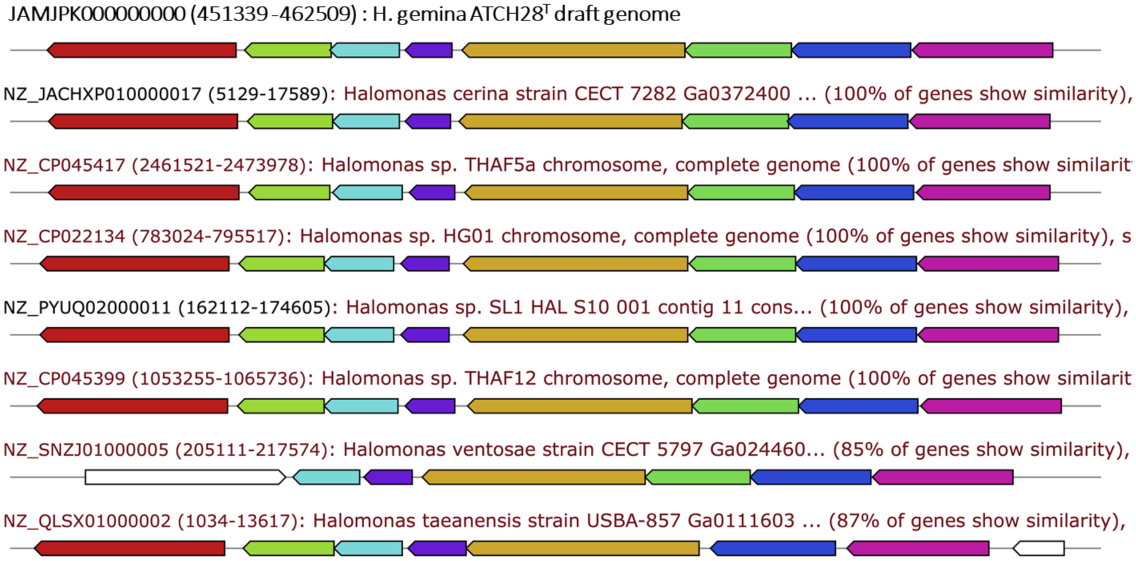

Figure S2. ClusterBlast results show, that up to five *Halomonas* strains share the same structure of DFOE biosynthesis gene cluster with strain ATCH28^{T} , while *H. ventosae* CECT 5797 and *H. taeanensis* USBA-857 have lower cluster similarity (85 to 87%) due to different gene components withtin this cluster. The accession number of each genome and location of each putative gene cluster are shown in brackets.

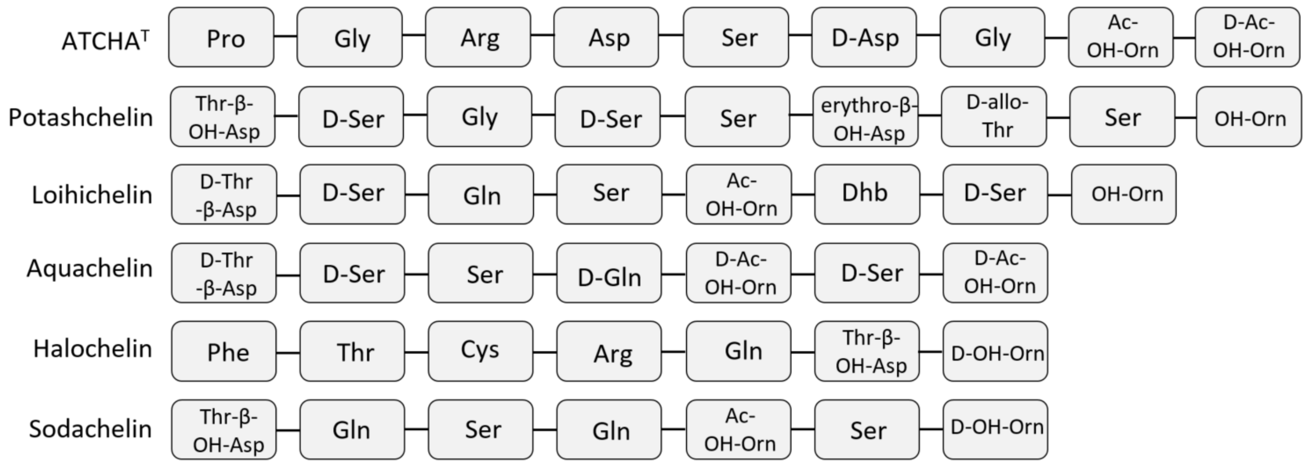

Figure S3. Amino acid composition of the siderophore produced by strain ATCHA^T (predicted by AntiSMASH), potashchelin (Li et al. 2020), loihichelin (Homann et al. 2009), aquachelin (Martinez et al. 2000), halochelin (O'mar et al. 2015) and sodachelin (Serrano-Figueroa et al. 2016).

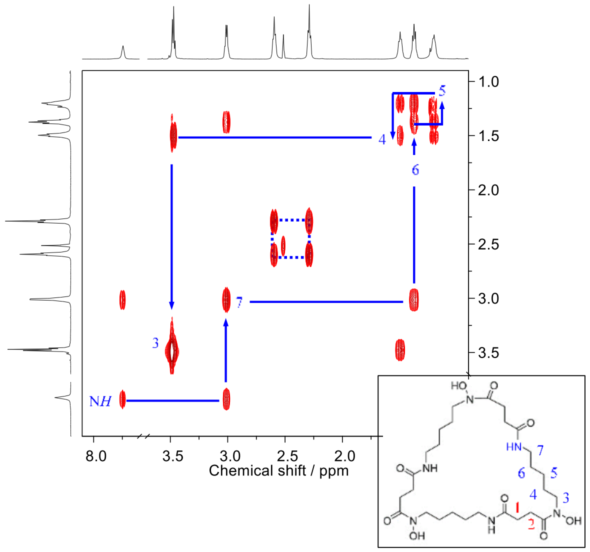

Figure S4. ^{1}H,^{1}H-correlation spectrum (COSY) of desferrioxamine E recorded at 14.1 T in DMSO-d_6 at 25 °C. In the COSY spectrum, the two isolated spin systems of the succinyl residue, methylene groups 1 and 2, (dotted square) and the *N*-hydroxy cadaverine residue, NH group as well as methylene groups 7 through 3 ("spin walk" indicated by arrows), are easily discernible.Signal 7 is unambiguously assigned to the methylene group adjacent to the amide group owing to their scalar spin-spin coupling. Since H7 shows coupling to both NH and H6, its associated signal appears as a partially overlapping doublet of triplets (pseudo-quartet), contrary to methylene group 3 that shows coupling only to adjacent methylene group 4 thus appearing as a triplet, cf. Figure 2B.

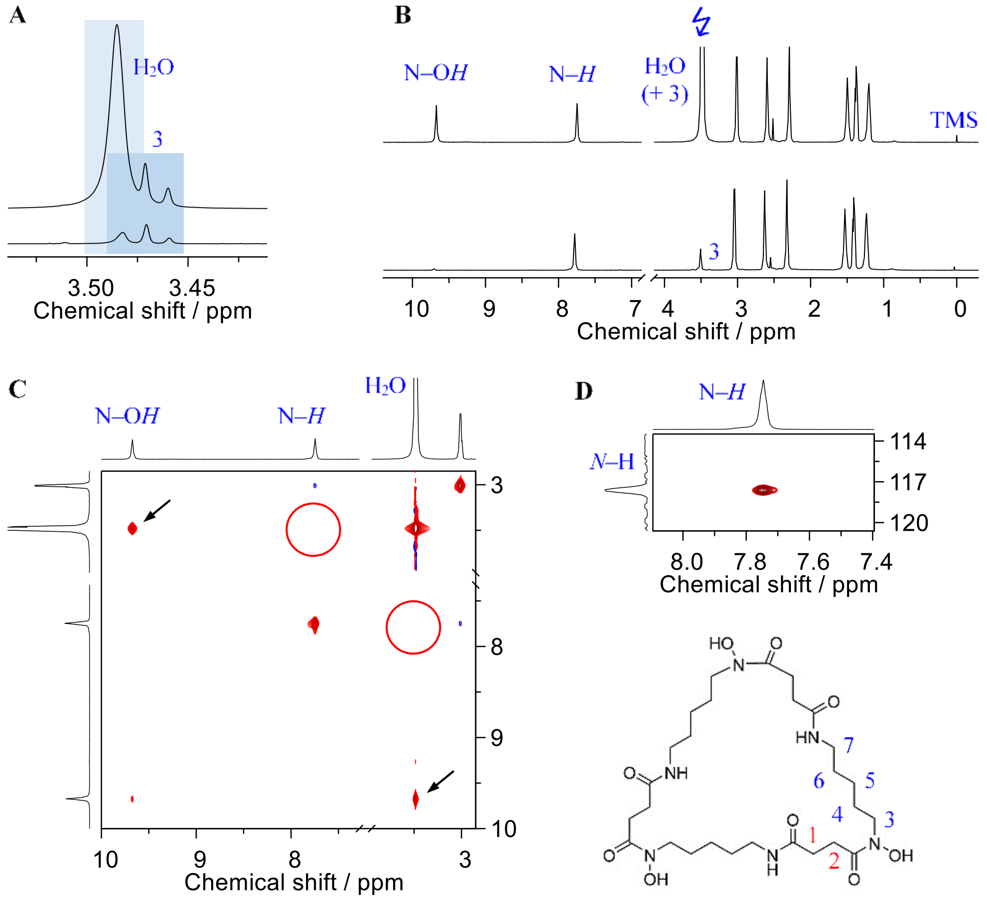

Figure S5. NMR spectra obtained from a solution of 25 mg of DFOE in DMSO-d_6. (A) ^{1}H NMR (top) and CPMG-^{1}H NMR spectrum (bottom) showing the region comprising the signals of CH_2 group 3 and water (in DMSO); (B) ^{1}H NMR spectrum without (top) and with application of a 2 s pre-saturation selective pulse on the water resonance; (C) H,H-ROESY spectrum, 200 ms mixing time; (D) ^{1}H,^{15}N-HSQC spectrum, 90 Hz opted for ^1J. The signal of the residual water (in DMSO) obscures the signal of CH_2 group 3. Therefore, a ^{1}H NMR spectrum using a Carr-Purcell-Meiboom-Gill (CPMG) pulse train was used to disperse the broad water resonance, revealing the comparably sharp CH_2 resonance with longer T_2 relaxation time (Fig. S2A). As an alternative approach for removing the water signal, a pre-saturation sequence was used (Fig. S2B, bottom spectrum). This resulted in two observations: (i) although somewhat truncated, signal 3 became visible, and (ii) along with the water signal, the signal associated with the ^{1}H of the hydroxamic group also disappeared. This is strong indication of protons exchange between water and this particular functional group. This process was evidenced by the in-phase off-diagonal correlation signals between the exchanging sites (black arrows), whereas the proton in the amide group reveals no (or only very slow) exchange reaction, hence no corresponding signals (red circles). The ^{1}H,^{15}N-HSQC spectrum proves that the signal at δ_H ~ 7.75 ppm is associated with the proton attached to the amide nitrogen (δ_N ~ 117.6 ppm).

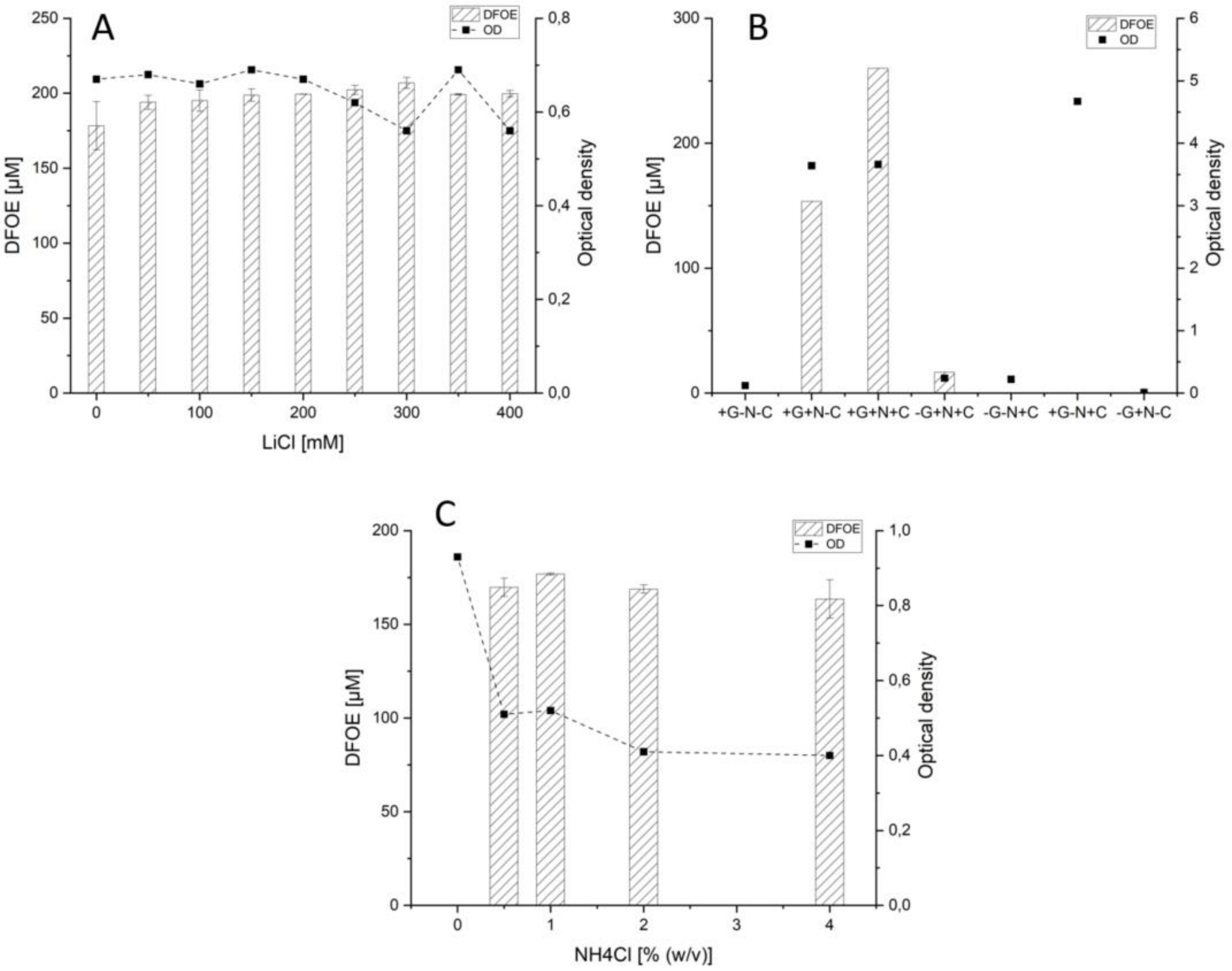

Figure S6. Effect of LiCl (A), Various combinations of carbon, and nitrogen sources (B) and NH_4Cl (C) on the production of DFOE in M9 by strain ATCH28^T; G, glucose; N, NH_4Cl, C, casamino acids; +, present in medium; -, absent in medium
